# A green route to prepare fluorescent and absorbent nano-hybrid hydrogel for water detection

**DOI:** 10.1038/s41598-017-04542-7

**Published:** 2017-06-29

**Authors:** Yiqiang Wu, Lijun Wang, Yan Qing, Ning Yan, Cuihua Tian, Yuanxin Huang

**Affiliations:** 1 College of Materials Science and Technology, Central South University of Forestry and Technology, Hunan, China; 2 Hunan Provincial Collaborative Innovation Center for High-efficiency Utilization of Wood and Bamboo Resources, Central South University of Forestry and Technology, Hunan, China; 3 Faculty of Forestry, University of Toronto, Toronto, Canada

## Abstract

An environment-friendly fluorescent nano-hybrid hydrogel has been synthesized successfully, from cellulose nanocrystal (CNC), acrylic acid (AA) and phosphorescent Eu^2+^/Dy^3+^ doped SrAl_2_O_4_ via free radical polymerization. The hydrogel network matrix fixed Eu^2+^/Dy^3+^ doped SrAl_2_O_4_ nanoparticles by polymer chains with coordinate bonds that prevented particles from aggregating and quenching in water. The fluorescent nano-hybrid hydrogel exhibited extremely high water absorption of which the swelling ratio in distilled water and NaCl salt solution were respectively of 323.35 g/g and 32.65 g/g. Furthermore, the hydrogel displayed excellent water retention property that can keep half of the moisture even exposed to 80 °C for 210 min. Besides, the hydrogel had bright green fluorescence under the sunlight or ultraviolet excitation, and the fluorescence intensity was up to 125477 after swelling 50 times in water. The time-resolved photoluminescence (TRPL) afterglow decay examination showed that the fluorescent emission persisted for 4 h after hydrogels excited at 368 nm wavelength UV-light for 10 min. The fluorescence intensity behaved significant linear relationship with the swelling ratio. As a result, these hydrogels were considered as promising candidates for the preparation of stable and sensitive sensor materials in green water detection.

## Introduction

Water and humidity detection is widely used in various fields, including water leakage detection, chemical reaction control, biomedical carrier and environmental monitor^1, 2^. There are tremendous efforts have been dedicated for the quantification and tracing water (or humidity) conductivity accurately and efficient in the past decades^3, 4^. It was demonstrated that the Karl Fischer titration is a representative way to detect water content^5^. This traditional approach can detect water content accurately, however, the long processing time and usage of toxic and smelly reagents are considered as the significant limitations. In order to eliminate toxic reagents in Karl Fischer titration method, Koupparls developed an improved method by replacing toxic pyridine^6^. The processing time consumption and low efficiency were reported as major obstruction. Currently, there are increasingly emerging other methods for water and humidity detection, such as microwave interferometry^7^, raman spectroscopy^8^, and solid phase extraction method^4^. These methods required complex and expensive equipments for better monitoring water conductivity. Therefore, a response sensitive, highly precious, environmental-friendly and efficient method for the detection of water and humidity is still of great challenge.

Fortunately, the emergency of optical transmission attracts numerous attentions for green and efficient water detection. Compared to the above-mentioned methods, there are several exceptional advantages in optical transmission, including high color purity and precision. The optical transmission involves many smart sensors which were majorly composed on the basis of fluorescent materials^9–12^. One of the representative fluorescent materials is synthesized by rare-earth, which has excellent fluorescent property^13–15^. In the past years, rare-earth fluorescent material was studied primarily on Eu^2+^/Dy^3+^ doped SrAl_2_O_4_ for the merits of environment-harmless and strong green fluorescence^16, 17^. It was reported that phosphorescent Eu^2+^/Dy^3+^ doped SrAl_2_O_4_ was successfully synthesized and used as temperature measurement^18^. However, Eu^2+^/Dy^3+^ doped SrAl_2_O_4_ has poor stability in aqueous solution and is easily quenched out particularly through water flow. As a result, the development of a substrate to load Eu^2+^/Dy^3+^ doped SrAl_2_O_4_ tightly in water is the fundamental way that the fluorescent materials are safely used in green water detection.

Considering the stability and water absorbent, hydrogels constructed by manes of physical or chemical bonds, are good candidate to be used as efficient substrates. The hydrogels can not only absorb large amount of water, but also keep water-saturated state for a long duration^19–22^. Besides, reactive groups on the polymer chains of hydrogels are able to form coordination bonds with metal elements, which in turns dispersed stably in the obtained hydrogels. Poly vinyl alcohol (PVA) was used as a precursor to synthesize intercrossed phosphorescent Eu^2+^/Dy^3+^ doped SrAl_2_O_4_ hydrogels to overcome the aggregation induced quenching effect^23^. According to the origin of raw materials^24^, hydrogels are generally classified as biopolymer-based and synthetic-based^25–27^. Referring to synthetic-based ones, the monomer of synthetic materials, such as vinyl acetate^28^ and acrylamide^29^, which are mainly manufactured as disposable products and disposed by landfill and incineration, leading to the serious environmental pollution. Thus natural polymers, such as polysaccharides, having non-toxicity, renewability, low-cost, biocompatibility, biodegradability, and abundant reactive sites, are desirable to replace synthetic polymers. Polysaccharides, including chitosan^30, 31^, hyaluronic acid^32^, cellulose^33–35^, and others^36^, were used to prepared hydrogels. Especially cellulose, which is the structural component of the cell wall of green plants, exhibits good affinity and sensitivity to water. Cellulose Nanocrystal (CNC) has abundant reactive –OH groups that can be grafted with polyacrylic acid (PAA), which is a material with excellent swelling capacities^37^. Owing to three-dimensional networks of hydrogels by physical or chemical cross-links^38^, cellulose-based hydrogels can absorb large quantities of water in short time^39^ and maintain water-saturated state. Due to these attractive structural properties, cellulose-based hydrogels are considered as the carrier to fabricate Eu^2+^/Dy^3+^ doped SrAl_2_O_4_ materials.

In the present work, we prepare cellulose nanocrystal hydrogels grafted with polyacrylate (CNC-g-PAA), in which phosphorescent Eu^2+^/Dy^3+^ doped SrAl_2_O_4_ was maintained stably and tightly by physical incorporation. The structure and properties of these hydrogels are investigated by wide angle X-ray diffraction (WAXD), fourier transform infrared (FT-IR) spectroscopy, scanning electron microscopy (SEM), transmission electron microscopy (TEM), fluorescence spectroscopy (FS), water absorption test, and water retention measurement. Interestingly, the fluorescence intensity for the as-prepared hydrogels shows a direct linear proportional relationship to water absorption amount with an excellent fluorescent stability. It is believed that these hydrogels are promising as green water sensors for the excellent sensitivity and stability.

## Results and Discussion

### Structure and miscibility analysis

**Figure 1:**
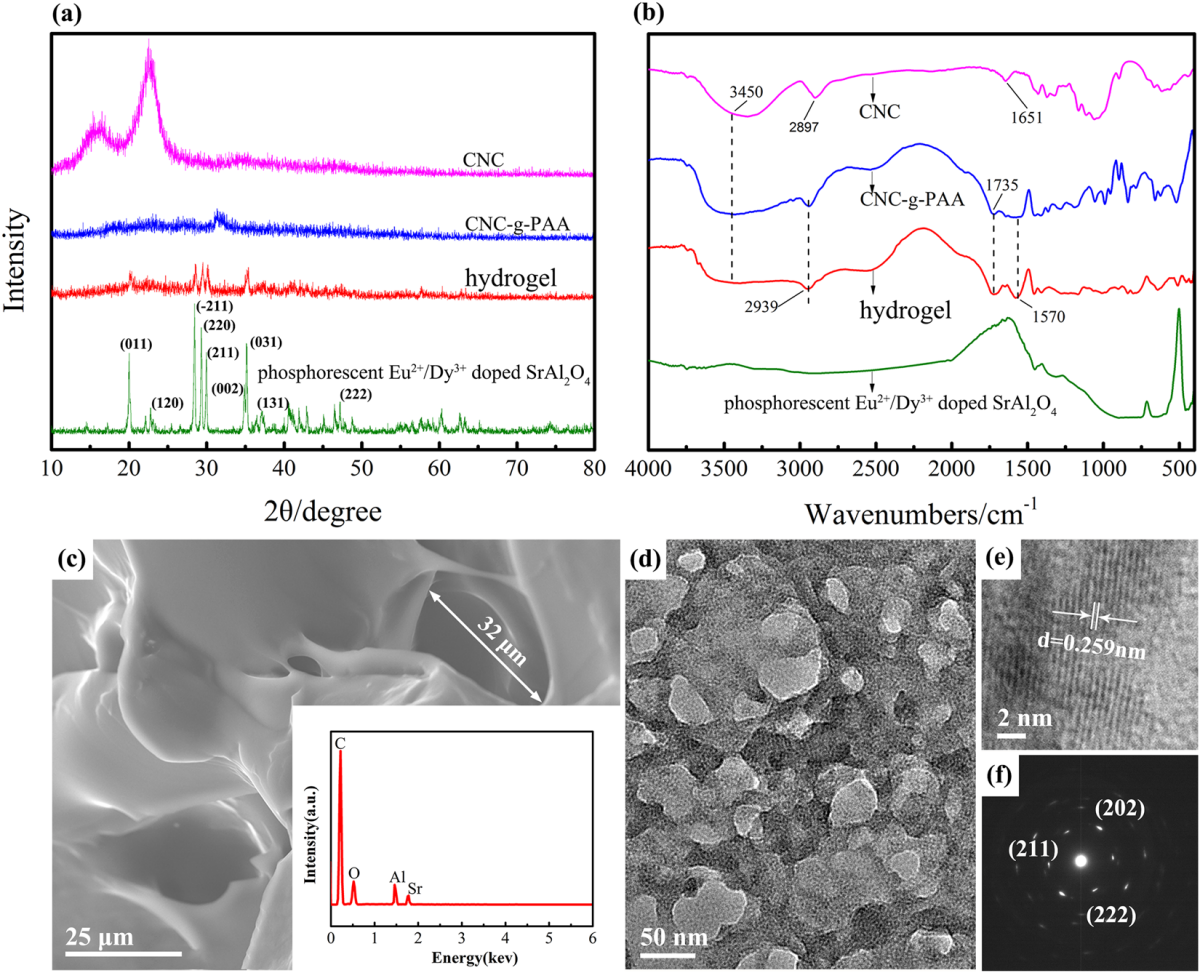
(**a**) Wide angle X-ray diffraction patterns of CNC, CNC-g-PAA, hydrogel and phosphorescent Eu^2+^/Dy^3+^ doped SrAl_2_O_4._ (**b**) FT-IR spectra of CNC,CNC-g-PAA, hydrogel and phosphorescent Eu^2+^/Dy^3+^ doped SrAl_2_O_4_. (**c**) SEM image of the hydrogel, the interleaved pattern in image (**c**) indicates the EDS spectrum of nano-hybrid hydrogel. (**d**) TEM image of hydrogel, it exhibits the phosphorescent Eu^2+^/Dy^3+^ doped SrAl_2_O_4_ in hydrogel decentralized state. (**e**) A typical crystalline HRTEM image of phosphorescent Eu^2+^/Dy^3+^ doped SrAl_2_O_4_ in the hydrogel. (**f**) The selected area electron diffraction pattern of phosphorescent Eu^2+^/Dy^3+^ doped SrAl_2_O_4_ in the hydrogel.

The structure and interaction between CNC-g-PAA and phosphorescent Eu^2+^/Dy^3+^ doped SrAl_2_O_4_ in the hydrogel were analyzed by XRD and FT-IR. Figure 1a shows the XRD patterns of hydrogel, CNC-g-PAA, CNC and phosphorescent Eu^2+^/Dy^3+^ doped SrAl_2_O_4_. The CNC samples exhibited main typical crystalline peaks at 2θ = 15.13°, 2θ = 21.12° and 2θ = 22.73°. After the graft polymerization of AA into CNC backbone chains, it was observed that all of these peaks almost disappeared in the XRD patterns of CNC-g-PAA and hydrogel. This suggested that all CNC participated in the graft polymerization as the AA graft polymerization in the CNC backbone chains would destroy the original crystallinity of CNC by leading to an amorphous pattern with a diminished peak intensity^37^. However, from phosphorescent Eu^2+^/Dy^3+^ doped SrAl_2_O_4_, it is clear that the sharp peaks of phosphorescent Eu^2+^/Dy^3+^ doped SrAl_2_O_4_ showed good crystallinity, indicating pure monoclinic phase diffraction peaks of phosphorescent Eu^2+^/Dy^3+^ doped SrAl_2_O_4_. The special peaks of phosphorescent Eu^2+^/Dy^3+^ doped SrAl_2_O_4_ could be observed in the patterns of the hydrogel. It was evident that phosphorescent Eu^2+^/Dy^3+^ doped SrAl_2_O_4_ was incorporated successfully into the cellulose hydrogels and the crystal structure of phosphorescent Eu^2+^/Dy^3+^ doped SrAl_2_O_4_ was well maintained in the cellulose matrix. Namely, there was almost no change in the structure and character of phosphorescent Eu^2+^/Dy^3+^ doped SrAl_2_O_4_ in the hydrogel.

The FT-IR spectra of pure CNC, CNC-g-PAA, phosphorescent Eu^2+^/Dy^3+^ doped SrAl_2_O_4_ and hydrogel are shown in Fig. 1b. The broad absorption peak at 3400-3500 cm^−1^ was relative to the intermolecular hydrogen bonding. The absorption bands at 2939 cm^−1^ and 2897 cm^−1^ were assigned to the vibration of C-H. As it can be seen from the CNC spectrum, the absorption bands appeared at 1651 cm^−1^ that was assigned to the –OH. In addition, compared to the spectrum of CNC (Fig. 1b), the new absorption bands at 1735 cm^−1^ (attributed to the stretching vibrations of C = O groups) and 1570 cm^−1^ (ascribed to symmetric stretching vibrations of –COO^–^groups) appeared in the spectra of CNC-*g*-PAA and hydrogel^33, 34^. Overall, the absorption bands of the CNC spectra were more similar to the spectra of CNC-g-PAA and nano-hybrid hydrogel. These evidences confirmed that the partially neutralized AA was grafted onto CNC to form CNC-g-PAA and nano-hybrid hydrogel. It was noted that the absorption of phosphorescent Eu^2+^/Dy^3+^ doped SrAl_2_O_4_ in FT-IR was weaker compared with organic groups, thus an insignificant effect of phosphorescent Eu^2+^/Dy^3+^ doped SrAl_2_O_4_ on CNC-g-PAA existed in the spectrum. These results suggested that phosphorescent Eu^2+^/Dy^3+^ doped SrAl_2_O_4_ was mainly physically incorporated into the CNC-g-PAA hydrogel, which was consistent with the XRD results.

### Surface morphology and microstructural analysis

To better investigate the network formation, the porous structure of the hydrogel was studied using SEM. Figure 1c shows the morphological characteristics of the hydrogel in the dry state. The hydrogel exhibited a porous structure, as was expected, which provided sufficient space to hold a large amount of water. The pore sizes were between 20μm and 32μm, which was consistent with its high equilibrium swelling ratio. Compositional analysis of the hydrogel by energy-dispersive X-ray spectroscopy (EDS) indicated that they were composed of C, O, Al and Sr elements (inset in Fig. 1c), where Eu and Dy were not effectively detected owing to a relatively low doping concentration and an insufficient resolution of EDS.

To demonstrate the microstructural information as well as property of hydrogel, we implemented the TEM/HRTEM morphological analysis. Figure 1d represents the TEM image of nano-hybrid hydrogel. It exhibited the spherical structure of phosphorescent Eu^2+^/Dy^3+^ doped SrAl_2_O_4_ nanoparticles that were immobilized in the CNC-g-PAA matrices with 3D networks through electrostatic attraction and physical interaction between CNC-g-PAA and phosphorescent Eu^2+^/Dy^3+^ doped SrAl_2_O_4_, as supported by the results from Fig. 1a and b. Figure 1e shows the typical crystalline HRTEM image of phosphorescent Eu^2+^/Dy^3+^ doped SrAl_2_O_4_ in nano-hybrid hydrogel. It shows that the interplaner spacing between the lattice fringes was 0.259 nm, which corresponded to the d spacing for the (222) lattice planes of the monoclinic SrAl_2_O_4_ system. Figure 1f shows the selected area electron diffraction (SAED) pattern of nano-hybrid hydrogel as depicted in Fig. 1e. It was consistent with the high crystallinity and ordered structure pattern in crystallography. The diffraction spots could be indexed as monoclinic phase with lattice planes (202), (211) and (222), which were in good agreement with the results of XRD. The above results showed that phosphorescent Eu^2+^/Dy^3+^ doped SrAl_2_O_4_ was incorporated in to the high absorbent hydrogel and its crystal structure was not changed. Thus, phosphorescent Eu^2+^/Dy^3+^ doped SrAl_2_O_4_ luminescent performance was not affected. It confirmed that the fluorescence high absorbent hydrogels preparation was successful, expanding the potential use of high absorbent CNC hydrogels.

### Spectroscopic analysis

**Figure 2:**
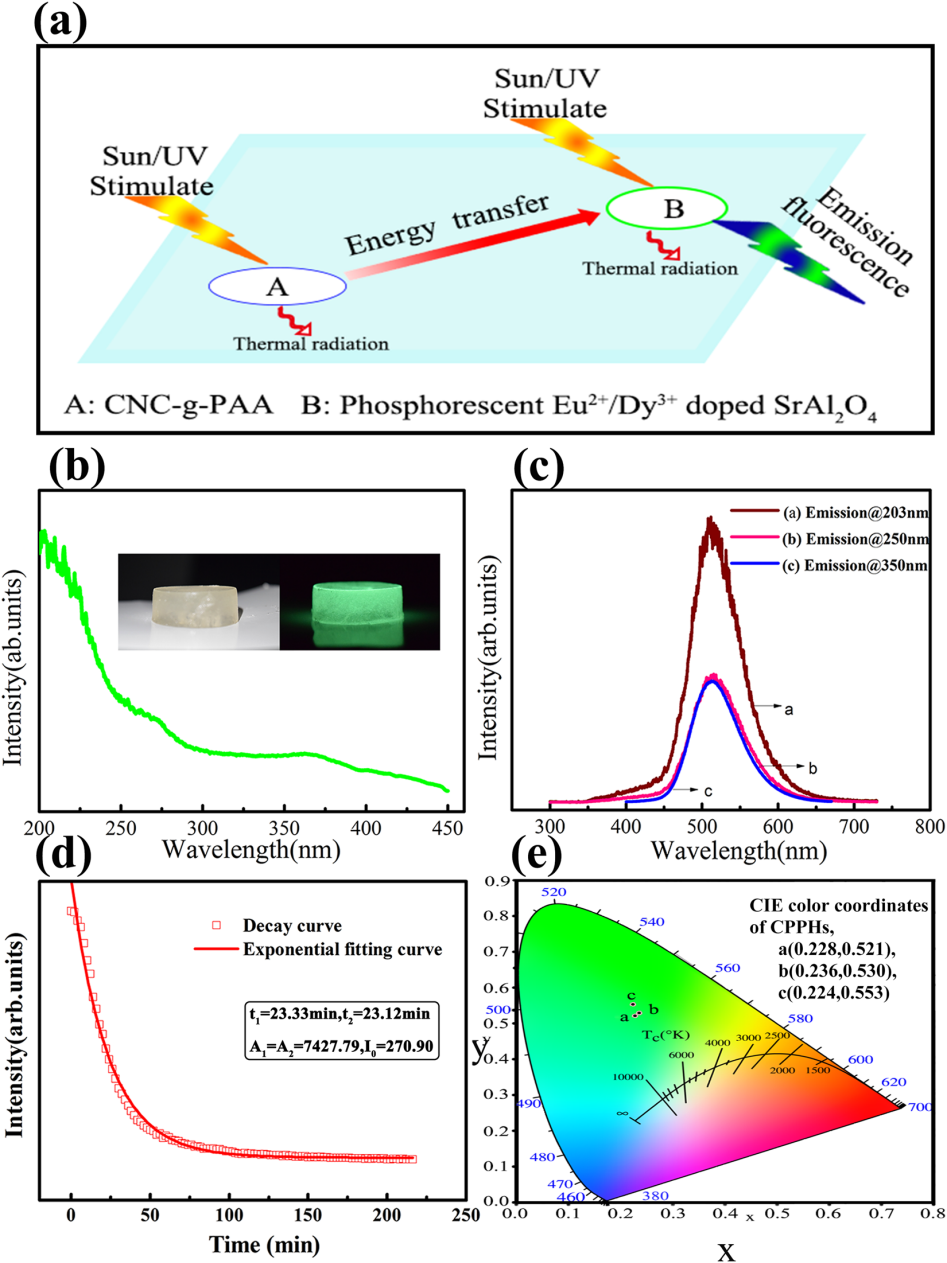
(**a**) Hydrogel luminescence mechanism. (**b**) The excitation spectrum of hydrogel at 513 nm emission wavelength; interleaved patterns show the photographs of hydrogel: the sample with yellowish white color before the excitation treatment of sunlight (the left one), the sample with strong green color after the excitation treatment of sunlight (the right one). (**c**) Photoluminescence spectra of hydrogel at different excitation wavelengths (λex = 203, 250 and 350 nm). (**d**) TRPL afterglow decay curve of hydrogel. (**e**) The photograph of CIE color coordinates of hydrogel.

Under the excitation of ultraviolet light or sunlight, hydrogel can generate an intense luminescence. The mechanism of photoluminescence is illustrated in Fig. 2a. Firstly, sunlight or ultraviolet light reaches the CNC-g-PAA base materials and CNC-g-PAA polymer absorbs sunlight or ultraviolet excitation energy, then it transfers the energy to phosphorescent Eu^2+^/Dy^3+^ doped SrAl_2_O_4_, which motivates phosphorescent Eu^2+^/Dy^3+^ doped SrAl_2_O_4_ to radiate fluorescence. The phosphorescent Eu^2+^/Dy^3+^ doped SrAl_2_O_4_, when excited by sunlight and ultraviolet, will emit fluorescence itself. Electron transition mainly happens in this emission process. First assume Eu^2+^ is converted to Eu^+^ and empty hole of the Fermi level lies between the level of Eu^2+^ and the middle of Valence band maximum. The hole that is released migrates through the valence band, is captured by Dy^3+^, which can be assumed that Dy^3+^ is then oxidized to Dy^4+^. When light irradiation stops, empty hole is motivated again and is released to the valence band. The hole migrates to the Eu^+^ and is captured and that results in producing fluorescence again, which is an important reason why light-emitting materials afterglow time lasts long^15, 17, 18^.

Figure 2b exhibits the excitation spectrum of hydrogel at 513 nm emission wavelength. It consists a single broad excitation band from 200 nm to 450 nm, presenting the maximum intensity peak at 203 nm. The excitation spectrum strongly overlaps with the UV–visible portion of solar radiation received at the Earth’s surface. This overlap signifies that the as-synthesized phosphor can be efficiently energized by a variety of illumination sources, especially, by the most common natural sunlight. The inset of Fig. 2b show the photographs of hydrogel: the sample with yellowish white color before the excitation treatment of sunlight (the left one), the sample with strong green color after the excitation treatment of sunlight (the right one).

Figure 2c emerged the emission spectra of the hydrogel at 203, 250 and 350 nm excitation wavelengths, which are all effectively the same differing only in their intensity. It attributes an emission at 511 nm wavelength and a maximum intensity is obtained for 203 nm excitation wavelength. Besides the intense photoluminescence spell it out, hydrogel also exhibits long lasting visible persistent luminescence after the removal of the excitation source. The time-resolved photoluminescence (TRPL) decay profile of the phosphor was recorded with the help of time correlated single photon counting technique. The TRPL decay curve and exponentially fitting curves with attenuation data parameter of nano-hybrid hydrogel monitored at 528 nm emission upon 368 nm excitation wavelength at room temperature are shown in Fig. 2d. The decay processes of phosphor possessed a double-exponential decay character. The decay behaviour of the phosphor can be fitted by an empirical equation stated as Equation(1)

1 $${\rm{I}}={{\rm{A}}}_{1}\exp (-t/{t}_{1})++{A}_{2}\exp (-t/{t}_{2})+{{\rm{I}}}_{0}$$

Where A_1_& A_2_ are the weighting constants parameters, t is the time period and t_1_& t_2_ are attenuation parameters for the exponential decay components. Using fitting functions, these parameters can be calculated by simulating the decay curves of hydrogel. The parameters generated from fitting are listed in the inset of Fig. 2d, The exponential fitting generated parameters are A_1_ = A_2_ = 7427.79, I_0_ = 270.90, t_1_ ~ 23.33 min and t_2_ ~ 23.12 min. It means, when the source lamp was switched off after 10 min, the intensity of the afterglow firstly decreases rapidly within 50 min and subsequently forms a stable long persistent emission more than 4 hours.

The CIE color coordinates obtained from emission spectra of hydrogel are A(x = 0.228 and y = 0.521), B(x = 0.236 and y = 0.530) and C(x = 0.224 and y = 0.553), respectively, corresponds to the emission spectra of hydrogel at 203, 250 and 350 nm excitation wavelengths, as shown in Fig. 2e, indicating the emission of the green color. This emission-color fine tuning along the CIE chromaticity diagram can be also modulated by chemical factors(Eu^2+^/Dy^3+^ concentration, nature of the electrons and holes) and physical parameters (excitation wavelength and temperature).

### Water absorbing swelling and water retention property analysis

**Figure 3:**
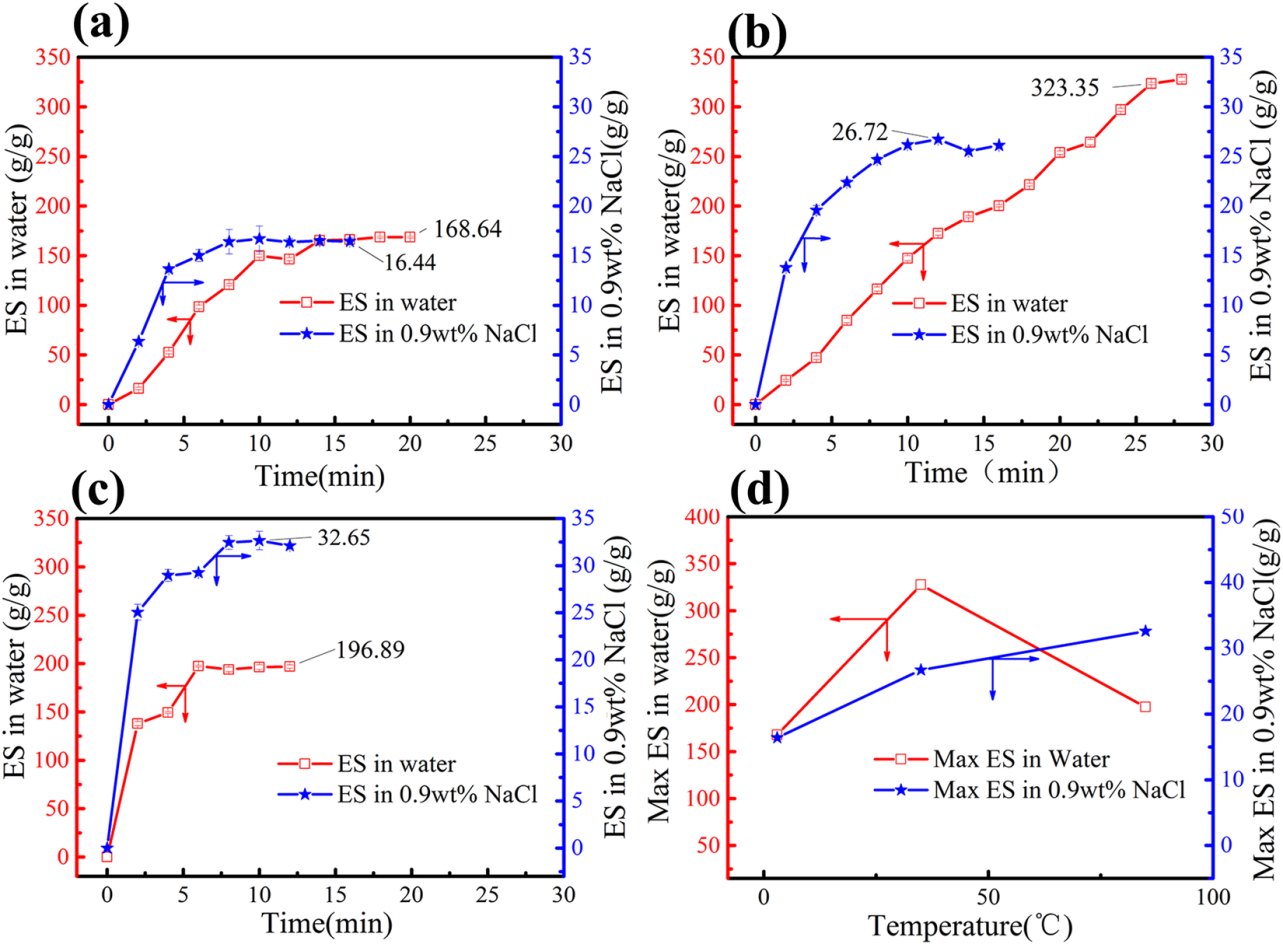
The temperature effect on the water absorbency, (**a**) 3 °C, (**b**) 35 °C, and (**c**) 85 °C (Data were given as means ± SD (n = 3)). (**d**) Max ES in water at 3 different temperature.

Characterization of the water-absorbing swelling ability of the prepared hydrogel is important for their potential use in different applications. The temperature and saline concentration impact on swelling ratio of the prepared hydrogel in distilled water and 0.9 wt% NaCl salt solutions was investigated and the results are shown in Fig. 3.

**Figure 4:**
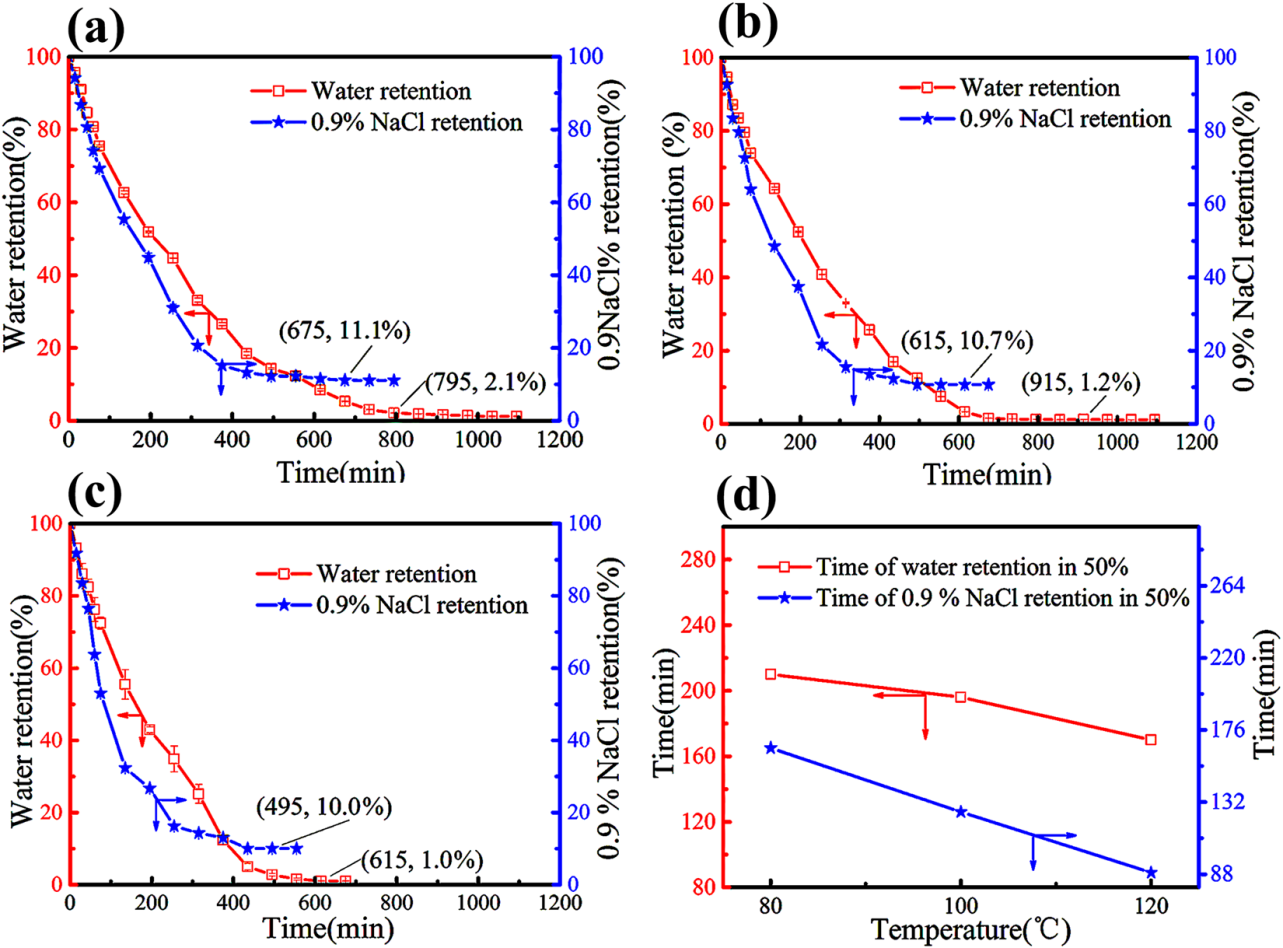
The hydrogel water retention behavior at (**a**) 80 °C, (**b**) 100 °C, and (**c**) 120 °C (Data were given as means (n = 3)). (**d**) Time of water retention in 50% at 3 different temperature.

Figure 3a–c are the swelling curves of the hydrogels at 3 °C, 35 °C and 85 °C, respectively. It can be seen from the figure that the swelling tendency of all hydrogels was basically the same. The first rapid swelling of the water, and then reduce the rate, and ultimately to achieve swelling balance. When the temperature was constant, the swelling rate and equilibrium swelling rate of this hydrogel in 0.9% NaCl solution was much lower than the swelling rate and equilibrium swelling rate in distilled water.

The decrease of the swelling capacity of the hydrogels is mainly because of the screening effect and a loss of the osmotic pressure difference between the hydrogels and the fluids^40^. In 0.9% NaCl salt solutions, the anion–anion rejection of the carboxylate groups of CNC is also obstructed by the Na^+^ ions that shield the carboxylate groups, so the swelling capacity is decreased. In addition, it can be seen from the figure that as the temperature increased, the rate of swelling of the hydrogel increased, and this property is independent of the ionic strength in the solution. According to Fig. 3d, the maximum swelling ratio of 323.35 g/g in distilled water and 32.65 g/g in 0.9 wt% NaCl salt solutions were obtained. In distilled water, the maximum equilibrium swelling increases first and then decreases after 35 °C. Due to hydrogen groups between hydroxyl and carboxyl in hydrogel network structure bonding with water molecules, and van der Waals force, the hydrophilicity of the hydrogel under low temperature was enhanced and the swelling became easy. As the temperature increased, the hydrophilic groups (carboxyl and hydroxyl groups) in the gel network structure were destroyed by hydrogen bonds formed by water molecules. At the same time, the hydrophobic interaction between the hydrophobic groups (alkyl groups) became stronger, resulting in a decrease in the swelling ability of the hydrogel. However, in salt solution, this effect will be weakened by strong ions, or even shielding. In conclusion, With the good absorption ability and the preference for the ambient temperature, the hydrogel application scope is expanding. This kind of strong fluorescent super absorbent nano-hybrid hydrogel can be applied to different temperature conditions for high water sensitivity detection.

It is important to evaluate the water retention behavior of highabsorbent in view of practical applications. In this work, the water retention of nano-hybrid hydrogel under various temperatures of 80 °C, 100 °C, and 120 °C was collected. As shown in Fig. 4a–c, the disappearing rate of Swelling of the hydrogel was different. At the beginning, the hydrogel had reached saturation due to the swelling equilibrium of the hydrogel. When it was put into the drying oven, a large amount of water began to drain, so the disappearing rate of swelling was very fast. As the moisture content in the hydrogel gradually decreased, it was difficult for the moisture to begin to become free from the hydrogel, so the rate of swelling became slow. When the water was completely lost, that is, when the sample was thoroughly dried, the disappearing rate of swelling of curve slope became zero. From the Fig. 4d, with the temperature increased, the time to water retention in 50% and 0.9% NaCl salt solutions retention in 50% decreased, this property was independent of the ionic strength in the solution. The fully swollen hydrogel in 0.9% NaCl salt solutions and distilled water could retain more than 50% of the absorbed water when subjected at 80 °C for 210 min and 156 min, respectively. This kind of excellent water retention properties at high temperatures enables the application of this novel hydrogel in high temperature conditions for water detection.

### Sensing properties for water

**Figure 5:**
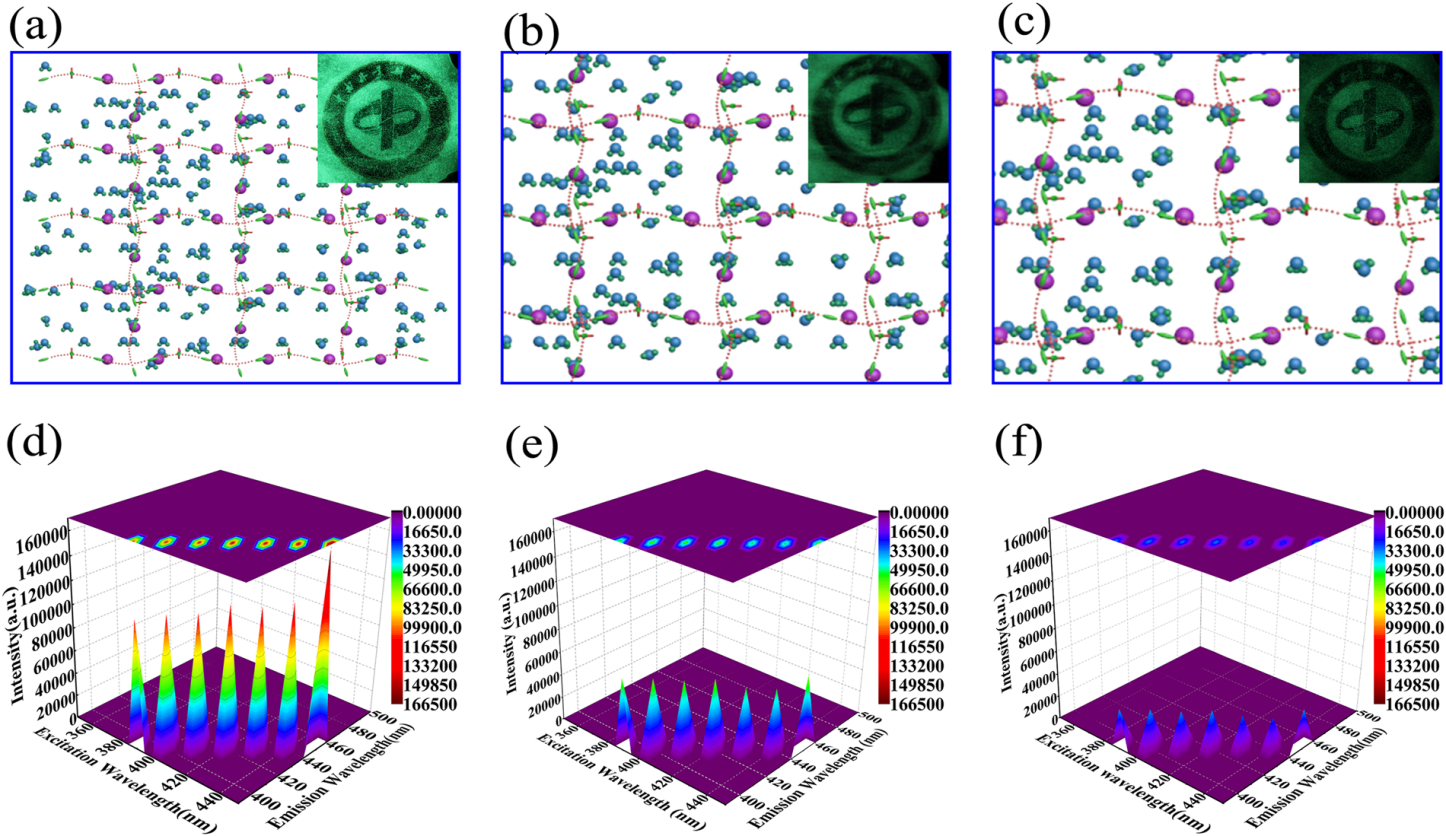
The structural model of hydrogel, inset shows the typical optical micrograph image of hydrogel of different swelling ratios respectively for 50 times(**a**), 100 times(**b**), 150 times(**c**). Excitation−emission maps of hydrogel absorbed the water (**d**) 50 times, (**e**) 100 times, (**f**) 150times and the corresponding contour maps.

Figure 5a–c show the structure and models for swelling ratios of 50 times, 100 times, 150 times of the hydrogel, respectively. From Fig. 5a–c it can be seen that with the increasing absorption of water, hydrogel internal hole structure increases. Figure 5a–c insets represent fluorescent images of hydrogel in different swelling ratios in dark condition.The greater the swelling ratio is, the lower the fluorescence intensity. According to the luminescent mechanism and the structure analysis, the energy transmission distance increases to result in the weakening of the fluorspar intensity. Photoluminescence (PL) spectra of hydrogel with various water contents are shown in Fig. 5d–f. All the photoluminescence spectra of hydrogel show the same form with exactly the same peaks, while the peak emission wavelength position increases with the increase in the excitation wavelength redshift. Meanwhile, the maximum emission wavelength showing a direct linear relationship with the corresponding excitation wavelength, i.e., the maximum emission wavelength, y, vs. the excitation wavelength, x, having the functional relationship of y = x. This kind of special linear relationship provides a foundation for its application in intelligent optical control and broadens the application fields of fluorescent high absorbent CNC hydrogel.

The peak value of fluorescence intensity of the hydrogel which absorbed water is 50 times higher than it own weight is 110362, 114415, 115907, 121487 and 125477, from picture’s left to right (Fig. 5d). When the weight of water absorption of hydrogel is 100 times than itself, the fluorescence intensity is 63108, 62827, 61444, 63042, 55754, 54817 and 66276 (Fig. 5e). And when the weight of absorbed water is 150 times higher than hydrogels’ own weight, the data of fluorescence intensity comes to 31415, 31321, 30732, 34794, 30042, 28367, 33937 (Fig. 5f). It can be seen clearly from the picture that as the water content of the hydrogel increased, the fluorescence intensity is decreasing gradually. Water absorption rate and fluorescence intensity has a linear relationship, so this kind of hydrogel could be used as fluorescent sensors for water detection, based on the change in fluorescence intensity.

### Exhibition of hydrogel stability

**Figure 6:**
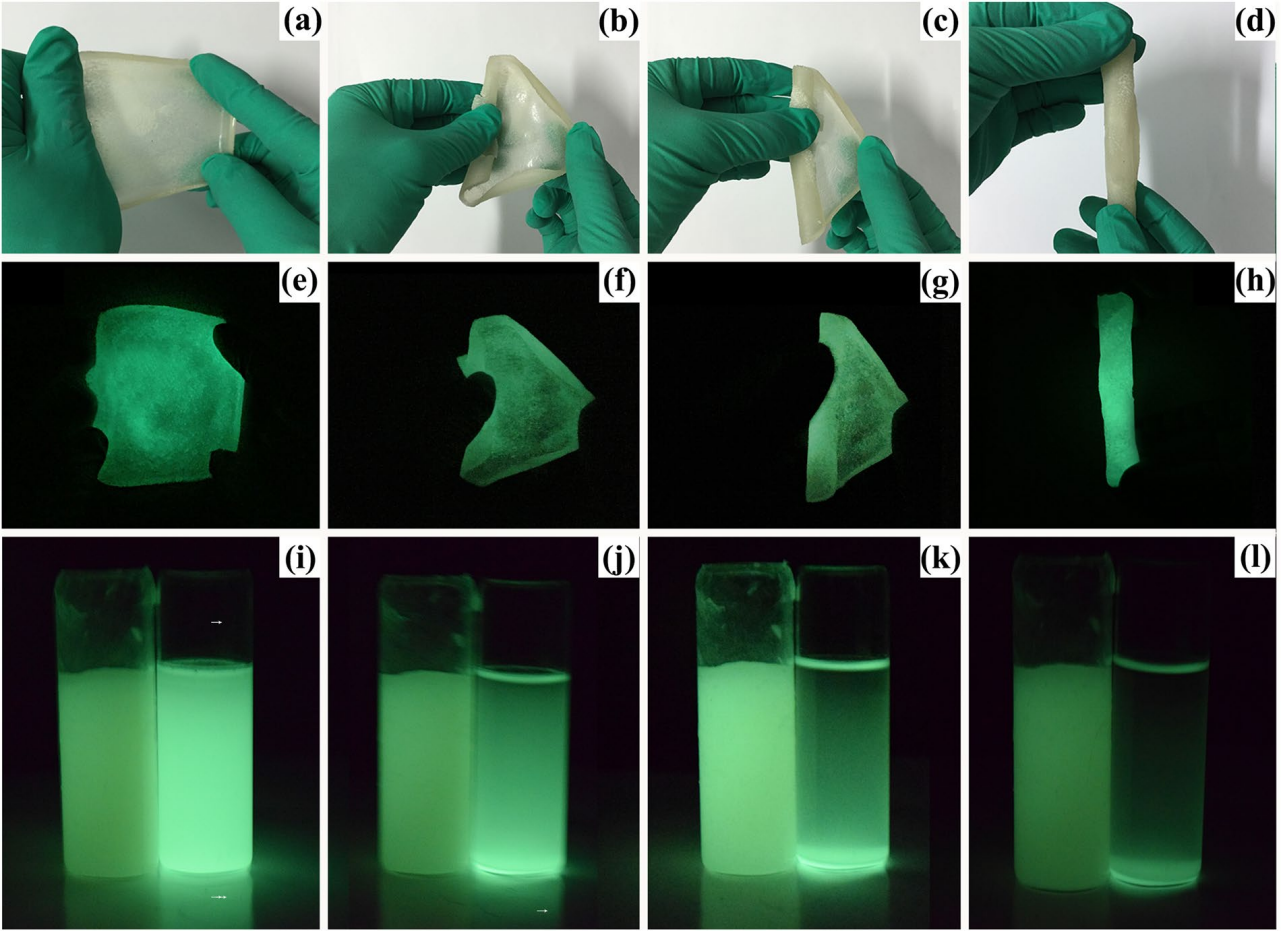
Optical image(**a–d**) indicates the high flexibility of hydrogel and optical image, (**e–h**) shows the fluorescence properties of hydrogel. Photographs of 0.32% aqueous solution of phosphorescent Eu^2+^/Dy^3+^ doped SrAl_2_O_4_ (right) and a water 100 times of hydrogel (left) under sunlight from 0 min (**i**), 5 min (**j**), 20 min (**k**) and 25 min (**l**) after mixing, respectively.

The as-prepared hydrogel was shown in Fig. 6. Figure 6a–d depicts the optical images of the hydrogels having high flexibility after several times folding. It can be folded up to 360° without any kind of deformation in the hydrogel. The Fig. 6e–h also shows the green afterglow emission of the hydrogel with the same fluorescence. The fold process doesn’t affect the fluorescence properties of hydrogel. A possible explanation is that the hydrophilic CNC-g-PAA high absorbent hydrogels matrix contributed to the good dispersion of phosphorescent Eu^2+^/Dy^3+^ doped SrAl_2_O_4_ in the aqueous solvent by preventing phosphorescent Eu^2+^/Dy^3+^ doped SrAl_2_O_4_ aggregation. The aggregation and precipitation of phosphorescent Eu^2+^/Dy^3+^ doped SrAl_2_O_4_ in water^17, 18^ can limit its application in the sensing field, while the hydrophilic CNC-g-PAA high absorbent hydrogels matrix could be used to improve its dispersion in water. Figure 6 shows the photographs of a 0.32 wt% aqueous dispersion of phosphorescent Eu^2+^/Dy^3+^ doped SrAl_2_O_4_ and a 100 times water swollen hydrogels after 0, 5, 20 and 25 min (Fig. 6i–l, respectively). Compared to the initial mixed dispersions (Fig. 6i), the phosphorescent Eu^2+^/Dy^3+^ doped SrAl_2_O_4_ particles precipitated even at 5 min (Fig. 6j right) and almost completely at 25 min (Fig. 6l right), whereas the hydrogel dispersion changed minimally (Fig. 6i–l left). Therefore, the dispersion of phosphorescent Eu^2+^/Dy^3+^ doped SrAl_2_O_4_ in aqueous solvent was improved significantly by using a CNC-g-PAA high absorbent hydrogels.

## Conclusions

In summary, strongly fluorescent and high absorbent nano-hybrid hydrogels were successfully constructed from cellulose nanocrystal, acrylic acid and phosphorescent Eu^2+^/Dy^3+^ doped SrAl_2_O_4_ via a chemical cross-linking process using N’,N methylenebisacrylamide. The phosphorescent Eu^2+^/Dy^3+^ doped SrAl_2_O_4_ particles were successfully embedded and dispersed evenly in the CNC-g-PAA hydrogel matrix. The original crystal structure and properties of phosphorescent Eu^2+^/Dy^3+^ doped SrAl_2_O_4_ were maintained in the hydrogels, leading to a strong fluorescence emission of hydrogel under sunlight and in the dark. The macroporous structure of the CNC-g-PAA hydrogels matrix not only protect the structure and character of the phosphorescent Eu^2+^/Dy^3+^ doped SrAl_2_O_4_ as the energy transmission base material, but also provide the pore wall as a high water absorbing and water retention material under the condition of different temperatures. Interestingly, the fluorescence intensity shows a direct linear proportional relationship to water absorption amount with an excellent fluorescent stability. Therefore, the hydrogel exhibited good potential for preparation of sensitive and stable sensor materials for water detection under the condition of different temperatures. Moreover, the hydrogel as renewable and biocompatible media have promise for broad applications, such as green water detection and biological imaging.

## Experimental Section

### Materials

The large cellulose fibers were provided by Shizuoka University, Japan. Phosphorescent Eu^2+^/Dy^3+^ doped SrAl_2_O_4_ was purchased from Hunan Institute for Non-Ferrous Metal Research. NaOH (AR grade) and Acrylic acid (AA,CP grade) were both bought from Tianjin Fuchen Chemical Reagents Factory (China). Potassium persulfate (AR grade), NaCl (AR grade), N’,N methylenebisacrylamide (MBA,CP grade), and Sulfuric acid (AR grade) were all supplied by Sinopharm Chemical Reagent Co., Ltd (China). Other chemicals used were available analytical grade reagents except otherwise noted.

### Synthesis of Strongly fluorescent nano-hybrid high absorbent hydrogels

**Figure 7:**
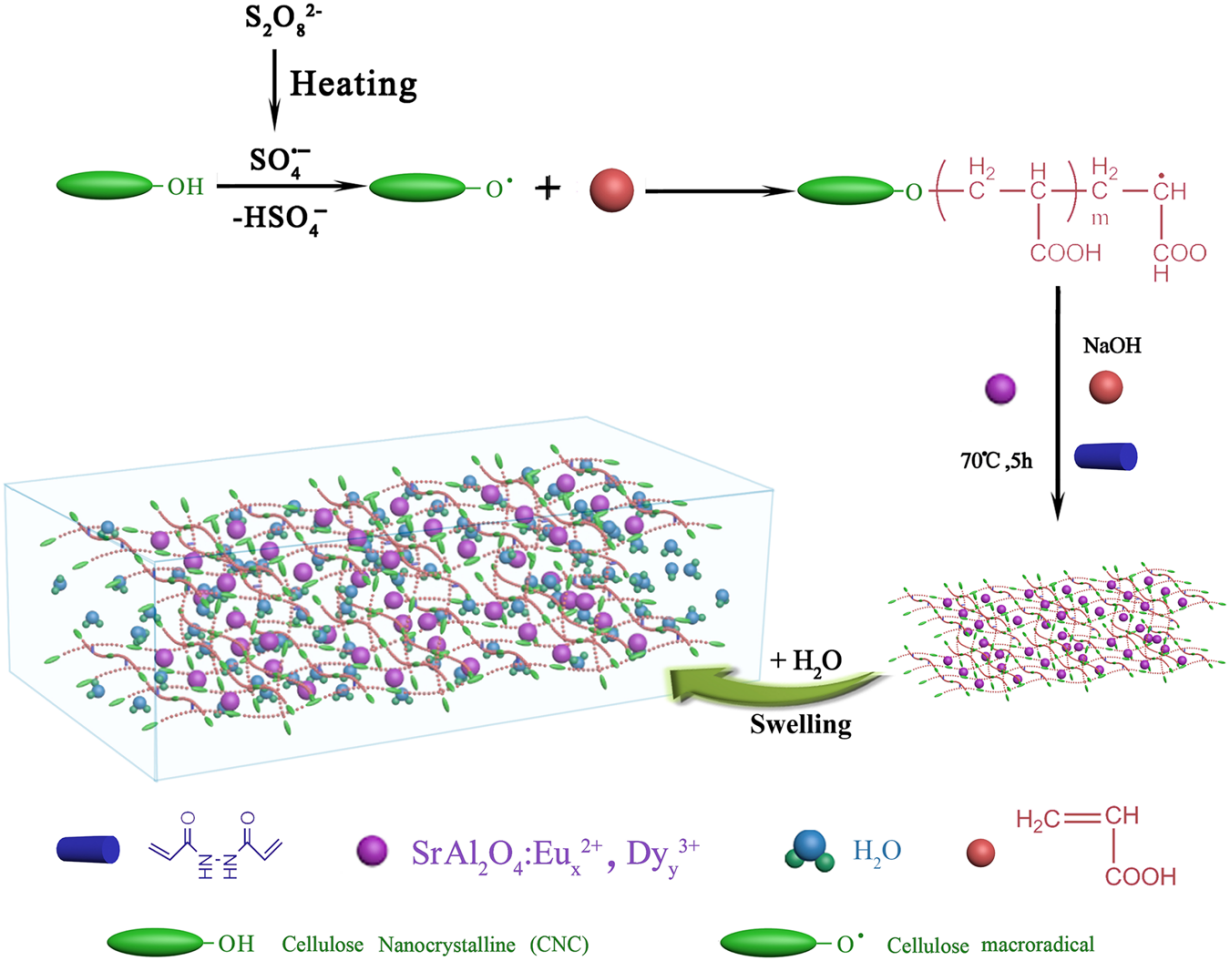
Schematic diagram of the synthesis of the nano-hybrid hydrogel.

The strongly fluorescent and high absorbent nano-hybrid hydrogels were prepared through radical polymerization. As shown in Fig. 7, acrylic acid was grafted on the CNC backbone in the presence of N’,N methylenebisacrylamide, potassium persulfate and phosphorescent Eu^2+^/Dy^3+^ doped SrAl_2_O_4_. The typical procedure was as follows: Firstly, 50 g 3.2 wt% CNC (obtained from large cellulose fibers hydrolyzed by 64 wt% sulfuric acid) and 0.08 g potassium persulfate were dispersed into 250 ml deionized water in water bath at 70°C for 20 minutes. Secondly, 50 g acrylic monomers were added into the mixture. Then, the mixture was added into 50 g acrylic acid along with 19 g NaOH, and 0.5 g phosphorescent Eu^2+^/Dy^3+^ doped SrAl_2_O_4_ gradually. The reaction system was stirred at 70°C for 5 hours to ensure the reaction complete. Finally, the hydrogel samples were obtained.

### Characterization

Prepared samples were characterized by wide angle X-ray diffraction (XRD). X-ray diffraction patterns of the nano-hybrid in scanning range of 10° ≤ 2θ ≤ 80° were obtained by using a X-ray diffractometer, china, equipped with a Cu Ka radiation source, operating at 40 kV and 40 mA. Functional groups of the nanohybrids were characterized via the FTIR spectrometer, (IRAffinity-1, Shimadzu, Japan). All samples were analyzed in the solid state as powder, adopting a KBr disk method, and then pressed into 1mm pellet. Spectra of the samples were recorded in the range of 4000 to 400 cm^−1^. Surface morphologies of the hydrogels were investigated by scanning electron microscopy (SEM, Hitachi SU-3500) equipped with energy dispersive X-ray spectroscopy (EDS, JEM-2100) to detect the elements. The microstructures of the hydrogel was studied by transmission electron microscopy (TEM, JEM-2010F) equipped with the selected area electron diffraction (SAED). Fluorescence spectra were recorded using a spectrofluorimeter (LS 55, Perkin Elmer, Inc., USA) and a photoluminescence spectrometer (FLSP-920, Edinburgh) equipped with a xenon lamp as an excitation source. The nano-hybrid hydrogels swelling measurements were done by immersing the nano-hybrid hydrogels in distilled water and 0.9 wt % NaCl solution at room temperature to measure its swelling, respectively. An accurately weighed sample (*M*_*0*_ = 0.1 ± 0.001 g) was immersed in two beakers, one filled with 500 mL distilled water and another one filled with 500 mL of salt solution. Then the swollen nano-hybrid hydrogels were taken out and the excess water was removed by a 100-mesh. The swelling ratio (ES) ^39^ was calculated according to the Equation (2):

2 $$ES=\frac{{M}_{1}-{M}_{0}}{{M}_{0}}$$

Where M_0_ (g) and M_1_ (g) are dry weight and swollen weight of samples, respectively. Water retention behavior of the synthesized sample was characterized as follows: Firstly, 10 g of fully swollen nano-hybrid hydrogel in distilled water were gently placed in 100 ml beakers. And then was put into ovens set at different temperatures i.e., 80°C, 100 °C and 120 °C. Finally, at a set time interval the sample was weighted. The nano-hybrid hydrogels retained water percentage (W_t_%)^39^was calculated using the Equation (3):

3 $${W}_{{\rm{t}}} \% =\frac{{W}_{t}}{{W}_{0}}$$

Where W_0_ stands for the mass of fully swollen hydrogel initially, and W_t_ is the mass of hydrogel after water loss at a certain interval of time. And this paper is in a data availability statement.
